# Moving from Vaccine Hesitancy to Acceptance: Engaging Underrepresented Employees in a Pediatric Academic Medical Center

**DOI:** 10.1097/pq9.0000000000000643

**Published:** 2023-04-10

**Authors:** Jean A. Connor, Francis Fynn-Thompson, James J. Horgan, Donna Luff, Patricia A. Hickey, Valerie L. Ward

**Affiliations:** From the * Cardiovascular, Critical Care and Perioperative, Patient Services, Boston Children’s Hospital, Harvard Medical School, Boston, Mass.; † Department of Cardiac Surgery; ‡ Center for Airway Disorders, Boston Children’s Hospital, Harvard Medical School, Boston, Mass.; § Office of General Counsel, Boston Children’s Hospital, Boston, Mass.; ¶ Immersive Design Systems, Boston Children’s Hospital, Harvard Medical School, Boston, Mass.; ‖ Sandra L. Fenwick Institute for Pediatric Health Equity and Inclusion, Boston Children’s Hospital, Boston, Mass.

## Abstract

Underrepresented populations historically underserved by the healthcare system and/or marginalized by systematic policies regionally and nationally were particularly vulnerable during the COVID-19 pandemic. While vaccine hesitancy has been described among hospital employees, the employees’ experiences from an underrepresented population cohort have not been reported. We, therefore, sought to understand employees’ vaccine experiences, hesitancy, and ways to enhance ongoing COVID-19 vaccine education and communication to build a hospital-wide culture of vaccine acceptance.

**Methods:**

We invited interprofessional staff from 5 clinical departments to participate in qualitative focus groups. Guiding questions were used to explore the experiences and perceptions of the staff. Using content analysis, we identified themes and recommendations for improvement.

**Results:**

We conducted 5 focus group sessions with over 50 participants. Four themes emerged; “Vaccine Fears Past and Present,” “Access to Information,” “Worries for Families,” and “Our Hospital is a Trusted Name.” Participants also provided recommendations for improvement in the messaging around the vaccine rollout. Consideration of how different employees access information, listening to staff needs, and recognizing the role of race and history were critical to engaging and improving the underrepresented employees’ vaccine acceptance.

**Conclusions:**

Exploring the concerns and fears of the COVID-19 vaccine within groups of underrepresented staff members through qualitative methods was key to understanding their vaccine hesitancy and implementing strategies to move toward vaccine acceptance in the hospital.

## INTRODUCTION

The impact of the COVID-19 pandemic on vulnerable, underserved, and underrepresented populations highlighted the priority of health equity in access to care and prevention.^1,2^ Early in the pandemic, racial and ethnic minorities in the United States had higher infection rates, hospital admissions, and death caused by COVID-19 than white, non-Hispanic people. The reasons for these findings were multifactorial and included racism, the presence of comorbidities, type of work, living in crowded conditions or heavily populated areas, and access to healthcare.^3–9^

As the newly developed COVID-19 vaccines became available, early reports of vaccine hesitancy were identified among hospital employees, particularly those underrepresented by race and ethnicity in the United States population.^10^ Vaccine hesitancy, defined as the delay in accepting or refusing vaccination despite vaccination services’ availability, is complex and context-specific to the time, place, and type of vaccine. In addition to well-described historical reasons, including race and ethnicity for vaccine hesitancy, it is also influenced by factors such as complacency or risk of contracting the disease, convenience or access to the vaccine, and confidence in vaccine effectiveness.^11–14^ For many hospital leaders attempting to safeguard their staff, a multipronged strategy was required to address vaccine hesitancy.^10^

During the early COVID-19 vaccination rollout in 2021, the COVID-19 task force at Boston Children’s Hospital (BCH) worked to understand the demographics of those hospital employees who were eligible and yet declined the vaccine. As SARS-CoV-2 evolved and increased across the country, the critical importance of maximizing the vaccination of healthcare workers became paramount. As we sought to increase employee vaccination, we recognized a gap in the literature and in planning our COVID-19 vaccination rollout by not first exploring possible COVID-19 vaccine hesitancy.

Therefore, we proposed using qualitative methods to engage hospital employees. Qualitative methods are valuable in providing insight into complex phenomena, exploring unique or unexpected events, understanding the experience of individuals with different stakes and roles, and giving those who are rarely heard a voice.^15–17^

## METHODS

### Context

BCH, an academic medical center, is a 454-bed quaternary care free-standing children’s hospital in an urban location in the northeast United States. The medical center consists of 2 main campuses and multiple regional satellites. It offers a complete range of healthcare services for infants, children, and young adults.

In the months leading up to the COVID-19 vaccine rollout, hospital leadership devised a strategy for vaccine education and how the vaccine would be made available for all employees. Hospital leadership facilitated a series of 1-hour virtual Town Halls for the vaccine education component. The recorded sessions were posted on the internal hospital website. Additionally, hospital leaders attended departmental and clinical staff meetings to provide information and answer questions. Upon the arrival of the vaccines, employees were grouped by role and COVID-19 exposure risk and offered days/times to receive the vaccine. Over 6–8 weeks, all groups were eligible to receive the vaccine.

### Qualitative Methods

**Fig. 1.**
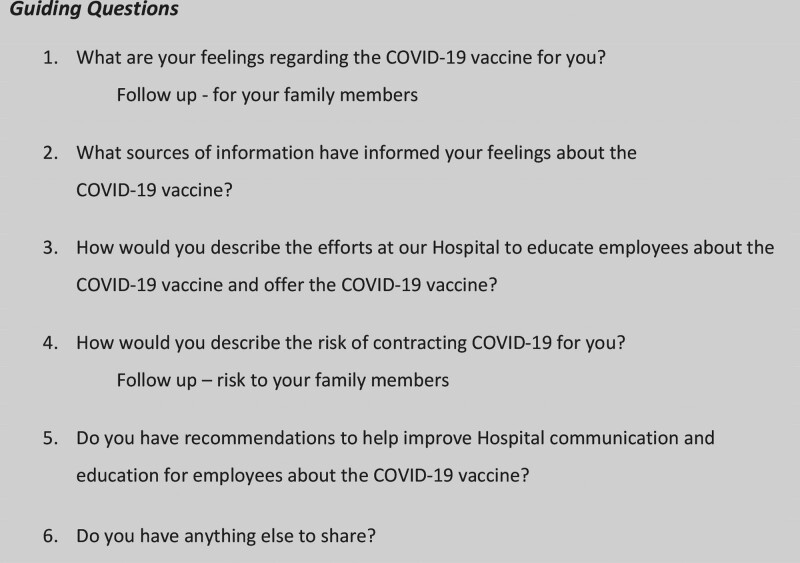
Guiding questions used in focus groups.

Following the hospital’s institutional review board policy for quality improvement activity, the study team members, which included COVID-19 subject matter experts, health equity and qualitative researchers, and surgical and clinical leaders, met to discuss both vaccine declination within the hospital and the available published literature regarding hesitancy and underrepresented populations/communities of color. The project leads used this discussion and literature to develop a set of questions to guide each focus group (Fig. 1). An informational flyer was developed at a 6th-grade literacy level describing the purpose and intent of the vaccine hesitancy focus groups and the contact information of the study team members to answer any questions about the study.^18^ Leaders of the hospital’s COVID-19 task force and the hospital’s Office of Health Equity and Inclusion reviewed and approved the flyer. Hospital leadership identified 5 areas across the organization for employee engagement, which were chosen based on the size of the clinical area, the mix of interprofessional staff, and higher declination rates of the vaccine. The project leads met with administrative leaders in these areas to provide information and answer questions. Administrative leaders used the flyer to inform and invite staff to join a focus group. For each of the 5 areas, the study team worked with administrative leaders to identify times during the workday that staff could voluntarily attend. All staff within these 5 areas were eligible to attend.

### Data Collection

The project leads (J.C. and F.F.T.) and a research assistant facilitated each focus group via Zoom over 3 weeks. These sessions were within 6 weeks of the rollout of the vaccine campaign. Each focus group, lasting no more than 1 hour, began with a short introduction reviewing the facilitators’ experience with qualitative inquiry at BCH and the sessions’ focus and intent. In the introduction, the facilitator emphasized that participation was voluntary, and there was no requirement to share their uncomfortable experience. Participants could leave the Zoom session at any time, it would not be recorded, and all information would be summarized as an aggregate and shared only with hospital leadership. At no time would the participants’ names or work areas appear in the dissemination of the information. The project lead (J.C.) utilized the semistructured interview questions to explore each area of focus. During the session, the research assistant and the project leads took notes, transcribing key statements/quotes of participants to support the theme development. Immediately after each session, the project leads and a senior study team member debriefed with each other. The project lead (J.C.) summarized the information gathered from each session and circulated it to study team members for review, further comment, and to guide data saturation.

### Data Analysis

The project leads then conducted a content analysis consisting of reading and rereading the data multiple times to understand the participants’ experience fully. It was during the reading of the written information that the coding process began. The project leads completed the first level of coding independently, identifying words or phrases participants emphasized, repeated, or stressed to facilitate importance. These codes were then compared between the project leads. After securing agreement, codes were clustered into categories and themes.^19,20^

To ensure the trustworthiness of the data analysis, the project leads set aside their thoughts and opinions to clearly hear what the participants shared. The project leads analyzed the data individually and then together to ascertain agreement of codes enhancing credibility, transferability, dependability, and confirmability of findings. The team maintained an audit trail of findings and decisions to ensure each step of the project could be traced, thus facilitating the replication of the study and confirmability of findings.^21^

**Fig. 2.**
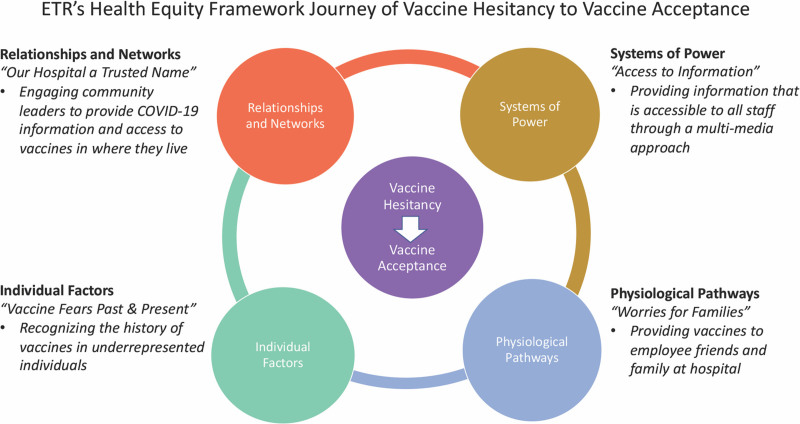
ETR’s Health Equity Framework Journey of vaccine hesitancy to vaccine acceptance.

We then utilized the Health Equity Framework by Peterson et al^1^ to highlight BCH leadership’s emerging themes, recommendations, and proposed actions (Fig. 2). Listening and using the information to improve or change is a proposed framework to address the inequities in the COVID-19 vaccine rollout in the United States.^2,22^ The study team generated a final summary report and presented this information to hospital leadership and employees in an open Town Hall meeting.

## RESULTS

### Demographics

We conducted 5 focus groups at various times throughout the day and evening to enable interdisciplinary staff participation. Over 50 staff members, 45 of whom were underrepresented employees (Black, Hispanic, and Asian), participated and represented a diversity of roles (registered nurse, clinical assistant, physician, pharmacist, social worker, radiology technician, respiratory therapist, pharmacist, pharmacy assistant, and environmental service). In addition, there was a mix of participants across the clinical areas who had received or declined the vaccine and those still deciding. Data saturation occurred after focus group 3; we then conducted 2 more focus groups to confirm the findings.

### Themes

**Fig. 3.**
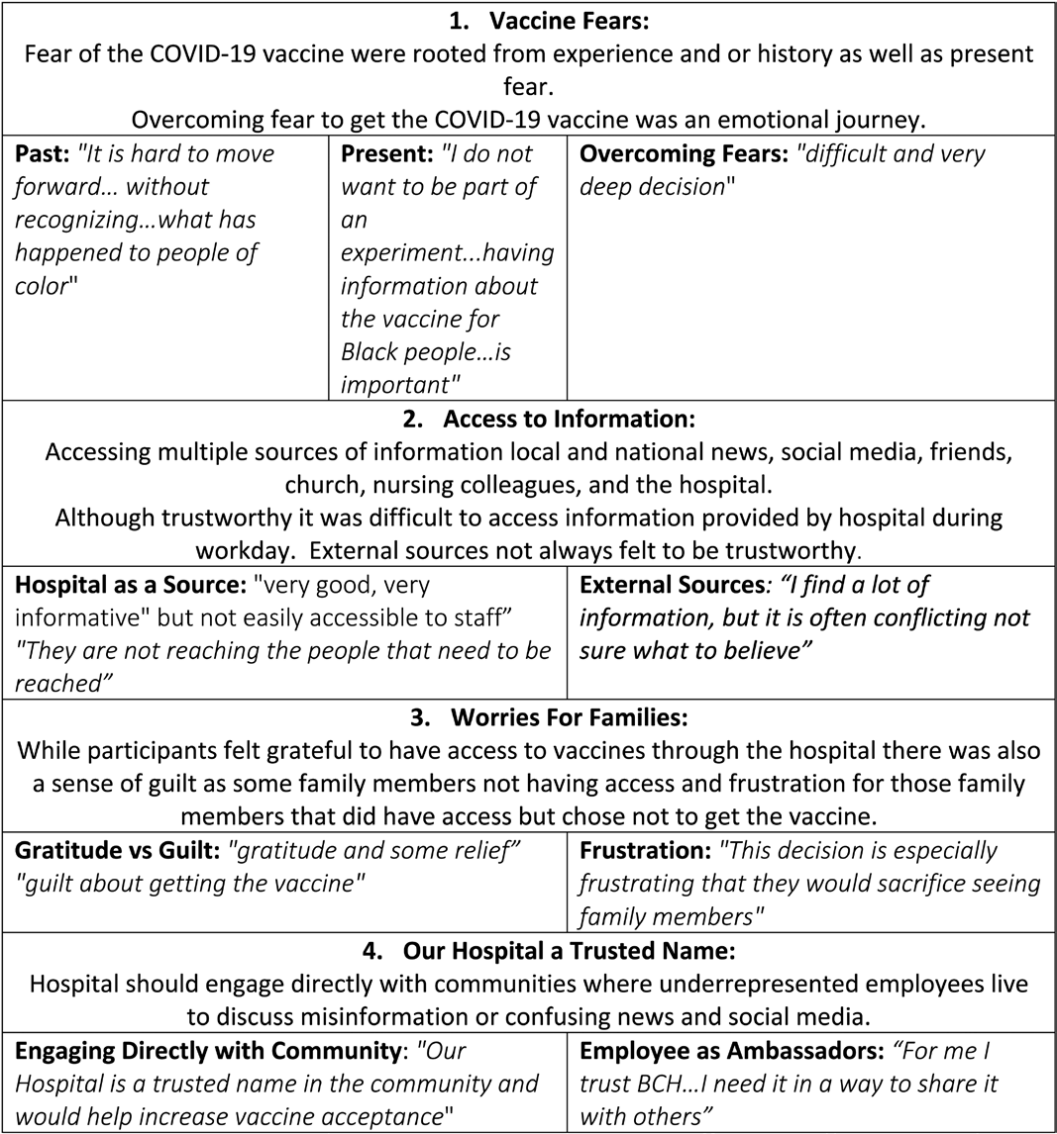
Vaccine hesitancy themes and subcategories.

**Fig. 4.**
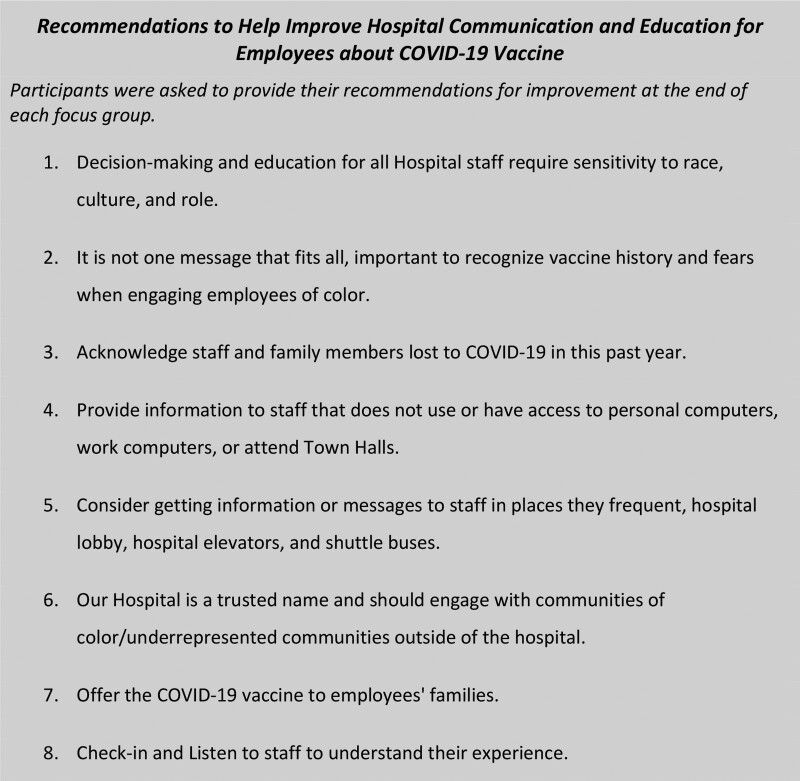
Recommendations to help improve hospital communication and education for employees about COVID-19 vaccine.

Four main themes emerged: “Vaccine Fears Past and Present,” “Access to Information,” “Worries for Families,” and “Our Hospital is a Trusted Name.” Within each theme, corresponding subcategories and supporting quotes were identified (Fig. 3). Participants also provided recommendations for improvement (Fig. 4). When viewed through the Health Equity Framework (Fig. 2), the 4 themes and recommendations produced actionable items based on participant recommendations.

### Vaccine Fears Past and Present

Participants from each focus group reported fear and concern about the COVID-19 vaccine, rooted in the history of vaccine deployment in underrepresented populations. One participant stated, *“It is hard to move forward… without recognizing…what has happened to people of color*.” Overall, participants agreed that while attention has been paid to national events in the media related to social injustice, BCH had not considered the impact of race and ethnicity on information disseminated related to the COVID-19 pandemic and the vaccine. They did not feel there was *“a place to express or anyone at [our hospital] to hear these fears*.” Participants in each session agreed that the focus groups were an important initiative for engaging employees and should have been offered earlier or before the vaccine rollout.

Several participants referred to the United States Public Health Service Syphilis Study at Tuskegee’^13,14^ as a historical example that generated fear and mistrust with medical institutions: *“Was this another time where people of color were being tested…?”* As one Black participant stated, *“I do not want to be part of an experiment...having information about the vaccine for Black people…is important.”* Participants in each of the focus groups reported feeling hesitant to receive the COVID-19 vaccine as “*it was new,” “made too quickly,” [and] “not enough known*.” In addition, these hesitant participants “*heard or saw*” several colleagues who had received the vaccine *“got sick and missed work unexpectantly*.” This consequence required other staff to fill in for their colleagues. One participant recalled overhearing another staff saying, *“Glad I did not get the vaccine; look what happened to them*.”

Other participant concerns were identified not related to race but to the COVID-19 vaccine. These concerns included the possible adverse side effects of the COVID-19 vaccine for staff who were pregnant or who might want to become pregnant, staff who had comorbidities, and the long-term effects of the vaccine. In addition, some vaccines hesitancy was due to *“long-standing concerns of svaccines in general… the connection to autism… the polio vaccine was rushed …people got sick*.”

Deciding to get the vaccine was based on several factors. One main factor was the fear of getting COVID-19, getting sick, and not caring for family members. Participants spoke about using their faith, church, family, and community members to guide their decision. For some, this was a *“difficult and very deep decision*,” and it was hard to “*overcome the history and the uncertainty*.” Some nonnursing participants discussed the COVID-19 vaccine with nursing colleagues as a trusted source of information, and *“this helped them with their decision*.” Another participant who had not received the vaccine stated they would risk receiving the vaccine *“For travel to another country to see family.”*

### Access to Information

Several participants cited accessing multiple sources of information to decide on getting the COVID-19 vaccine. These sources include local and national news, social media, friends, church, nursing colleagues, and the hospital. Most participants believed the information provided to them through BCH’s Town Halls was important, but there was “*not a message for them*.” For many, starting from a place of fear and not recognizing the history of race with vaccines was difficult. It was also difficult to access information for those who did not have a home computer or utilize a hospital computer to carry out their work responsibilities. Participants believed that BCH’s Town Halls were “very good, very informative” but not easily accessible to staff. Several participants stated*, “They are not reaching the people that need to be reached” … “The Town Halls were not easily viewed during work.” “To access the recording, you need VPN [virtual private network], which many employees do not have.”* Some focus group participants who had volunteered to help the hospital create information on the COVID-19 vaccine, educate staff in one-on-one conversations, and distribute the vaccine verbalized frustration, “*Not being able to move colleagues and family past vaccine hesitancy*.”

### “Worries for Families”

Staff reported feeling *“gratitude and some relief”* after getting the vaccine but worried about their family members getting COVID-19. Some participants also felt *“guilt about getting the vaccine”* and were *“frustrated”* that the vaccine was unavailable to the wider community. While some participants received the vaccination, some eligible family members did not receive it due to history and fear. One participant said, *“This decision is especially frustrating that they would sacrifice seeing family members.”* Participants believed if BCH could offer the vaccine to family members, it would be *“very positive… BCH cares about families, and that my family members trust BCH and would be less hesitant.”* Another participant believed recognizing employees and family members who have died from COVID-19 would be important, “*It is one year since the country shut down due to COVID-19 and people started to die. I believe this would please and elevate staff’s spirit.”*

### Our Hospital Is a Trusted Name

Participants believed BCH could and should engage directly with communities where underrepresented employees live to discuss misinformation, confusing news, and social media. As stated by one participant, *“Our Hospital is a trusted name in the community and would help increase vaccine acceptance*.” Also, several participants stated the importance of providing information directly to individuals, as many sources of information are not trustworthy or easily accessible. As one participant stated, *“For me, I trust BCH…I need it in a way to share it with others.”*

### Recommendations for Improvement

Participants offered recommendations for improvement at the end of each focus group (Fig. 3). Most importantly, it was recognized that information and education for staff are not one-size-fits all. Consideration of access to information, listening to employees’ needs, and recognizing the impact of race and history were critical to engaging and improving vaccine acceptance.

## DISCUSSION

This qualitative evaluation provides rich detail and adds to the current knowledge of COVID-19 vaccine hesitancy experienced by hospital employees during a global pandemic. Using focus groups offered a way to engage, listen, and understand the experiences and perceptions of vaccine hesitancy in underrepresented staff.^15,17^ Participants believed that these sessions were vital to verbalize their past and current experiences and fears and should have been offered before the hospital’s vaccine rollout. Of the hesitancy experienced by our participants, confidence in the COVID-19 vaccine seemed to be the most common factor. As defined in the WHO vaccine hesitancy model, confidence is the trust in the effectiveness and safety of vaccines, the system that delivers them, and policymakers’ motivations.^23^ Of note, this finding was important to consider, as one of the key focuses of the vaccine rollout education was the safety and effectiveness of the available COVID-19 vaccines.

We believe the intersection of the COVID-19 vaccine and race influenced this lack of confidence experienced by our employees. We learned through this assessment the importance of recognizing and acknowledging our employees’ race, past experiences, the history of race and tensions with medical systems, and their fears in the current context. Unfortunately, our underrepresented employees interpreted this lack of acknowledgment as not being seen or considered.

Another influence that impacted vaccine confidence was access to information. For many participants, accessing information during the workday was difficult. Most were involved in patient care and did not have a way to access the information after leaving the workplace. For other participants, accessing a work computer was not part of their role, and the perception would be that they were not working if they used a hospital computer during the workday. As a result, participants relied on other information sources, which caused confusion, fear, and vaccine hesitancy. These findings emphasize the need to meet employees where they are and provide accessible, meaningful, and actionable information.^10,18,24^ The COVID-19 pandemic has highlighted racial inequity and digital access inequity.^17^ In response, hospital leadership and the marketing team created ways to bring information to employees in different forums and areas within the hospital areas they frequent. We also implemented a “vaccine ambassador” program; to include trusted staff members from underrepresented groups who are available to clarify misconceptions regarding the vaccine; and low-tech communication, such as posting signs in multiple languages.

The Robert Wood Johnson Foundation states that “health begins where we live, work and play”^15^; hence, addressing vaccine hesitancy in healthcare employees at work and in their respective communities was essential to supporting a vaccine acceptance culture. While participants described difficulty accessing information from hospital sources on COVID-19 and the vaccine, there was trust in the information provided by the hospital. Hospital leadership recognized this as an opportunity to highlight the ongoing efforts of clinical staff working within communities. The hospital leadership also made vaccines available to friends and families of all employees. This intentional effort to provide vaccines for the hospital’s local community was well aligned with BCH’s declaration on equity, diversity, and inclusivity to anchor the institution in the community and be a part of sustainable changes that will result in more equitable health outcomes.^25^

While the information gained through this approach was meaningful and actionable, several limitations exist. We conducted all focus groups during the work hours of the employees. Participants used hospital computers; staff members shared computer stations with limited video capability in some sessions. As a result, the number of participants may be higher than stated. This study is a single-center evaluation, and our small sample size may limit the transferability of findings. Potential biases of how clinical areas were selected, the recruitment of employees, and the limited demographic information collected may have impacted the information received, and how these data were interpreted.

## CONCLUSIONS

The COVID-19 pandemic has challenged hospitals to provide clinical care to patients and ensure employee health and safety. Unfortunately, the distribution of the COVID-19 vaccines, while critical to ensuring hospital employee health and safety by many staff, was not universally accepted. Exploring the concerns and fears of the COVID-19 vaccine through qualitative focus groups in underrepresented staff was key to understanding vaccine hesitancy and creating steps to this latest phase of our COVID-19 vaccination acceptance journey.

## ACKNOWLEDGMENTS

Assistance with the study: We acknowledge Julia Koehler, MD, PhD, Zachary DiPasquale, MHA, and Shannon Engstrand, MPH, for contributing to this project.

## DISCLOSURE:

Dr. Ward is the Co-Leader of the Health Equity Core and Health Equity Advisor for the Children and Youth with Special Health Care Needs Research Network (CYSHCNet). This program is supported by the Health Resources and Services Administration (HRSA) of the U.S. Department of Health and Human Services (HHS) under UA6MC31101 CYSHCNet. This information or content and conclusions are those of the author and should not be construed as the official position or policy of, nor should any endorsements be inferred by HRSA, HHS, and the U.S. Government. Dr. Ward is also a member of the National Project Advisory Committee for a project being conducted by the Institute for Patient- and Family-Centered Care and Cincinnati Children’s Hospital Medical Center funded by the Lucile Packard Foundation for Children’s Health.

Drs. Connor and Fynn-Thompson are co-first authors.
